# Infestation Patterns and Climate-Based Projections for European Spongy Moth (*Lymantria dispar*) in Whirlpool Forest, Ontario, Canada

**DOI:** 10.3390/biology14111506

**Published:** 2025-10-28

**Authors:** Xiaolong Guo, Qianqian Wang

**Affiliations:** 1 Department of Earth and Environmental Sciences, St. Francis Xavier University, Antigonish, NS B2G 2W5, Canada; x2024ghb@stfx.ca; 2 China Conservation and Research Center for the Giant Panda, Chengdu 610057, China

**Keywords:** biodiversity, defoliation, insect defoliators, pest management, species-specific susceptibility

## Abstract

This study investigates spongy moth (*Lymantria dispar*) infestation patterns in Whirlpool Forest, Ontario, offering a region-specific perspective while largely corroborating existing findings. We analyzed egg mass distribution across 43 sampling plots, relating it to tree characteristics. Results revealed a preference for red oak species, with significant egg-laying above one meter. Positive correlations were found between tree diameter and egg mass quantity (*ρ* = 0.458, *p* < 0.001 above 1 m; *ρ* = 0.218, *p* = 0.006 below 1 m). Tree health was significantly associated with egg mass presence (*χ^2^* = 6.08, *p* = 0.014). A climate-based regression model (*R^2^* = 0.714, *p* < 0.05) projected substantial increases in outbreak area by 2100, with the most severe scenario predicting 9,927,378.49 hectares at risk. Sensitivity analysis showed a 1 °C temperature increase could expand the outbreak area by 814,100 hectares. These findings underscore complex infestation dynamics, challenging simplified models and emphasizing the need for tailored, adaptive forest management strategies in response to changing environmental conditions and pest behaviors.

## Simple Summary

The spongy moth is an invasive insect that damages forests across North America by eating tree leaves. We studied how these moths choose which trees to lay their eggs on in Whirlpool Forest near Niagara Falls, Ontario. We counted moth egg clusters on different tree species and measured tree sizes and health conditions. Our results showed that spongy moths strongly prefer oak trees, especially larger ones, and tend to place their eggs higher up on tree trunks. We found over 2000 egg clusters per hectare, which suggests moderate tree damage is likely in the coming growing season. Using climate data from the past eight years, we created a computer model to predict future moth outbreaks. The model shows that warmer temperatures and changing rainfall patterns could dramatically increase outbreak area across Ontario by the year 2100. In the worst climate scenario, the area at risk could expand from current levels to nearly 10 million hectares. These findings help forest managers understand which trees are most vulnerable and prepare for larger outbreaks as climate continues to change.

## 1. Introduction

Forest health is essential for maintaining ecosystem complexity and meeting human needs, integrating utilitarian and ecological measures of forest condition and functioning across multiple spatial scales. North America forests provide ecological services such as carbon storage, biodiversity, and climate regulation, as well as energy, construction materials, and sustenance for humans, thereby benefiting society on all scales, from the local to the global [1,2,3]. In recent years, factors such as climate change, fire, air pollution, fragmentation, and native and invasive pests have affected forests worldwide [1,2].

The European spongy moth, *Lymantria dispar* (Lepidoptera: Erebidae), is one of North America’s most damaging insect defoliators [4,5,6,7,8]. Late in the 1860s, this Eurasian moth was introduced to the United States to be fostered and reared, and subsequently expanded to invade forests across the United States [4,5,6]. Spongy moths were first observed invading Ontario, Canada around the 1970s and causing defoliation in large, forested areas of southern Ontario [6,9]. The spongy moth, a non-native pest, has been known to cause extensive defoliation in North American forests, leading to severe ecological and economic impacts [10]. According to Forest Health Conditions in Ontario 2021 [11], moderate-to-severe spongy moth infestations in Ontario reached 1,779,744 hectares, more than tripling the infestation area compared to 2020.

Defoliation from spongy moth outbreaks reduces the vigor and general health of forest and shade trees, making them vulnerable to attack by secondary lethal agents [12]. About 500 species of trees, primarily hardwoods such as oaks, are defoliated by spongy moths, making them susceptible to secondary attacks by other insects and pathogens that destroy the trees [6,8,9,13,14,15]. A five-year study in central Pennsylvania revealed that the spongy moth prefers chestnut oak (*Quercus prinus*) the most, followed closely by other oaks (*Quercus* spp.) and aspen (*Populus tremuloides*), while showing less preference for hardwoods like yellow-poplar (*Liriodendron tulipifera*), yellow birch (*Betula alleghaniensis*), black locust (*Robinia pseudoacacia*), striped maple (*Acer pensylvanicum*), ash (*Fraxinus* spp.), and black gum (*Nyssa sylvatica*) [16]. However, in a recent study it was also demonstrated that the spongy moth exhibits a stronger preference for Turkey oak compared to European beech and hornbeam, highlighting the importance of species-specific management strategies in forest conservation [17].

Control strategies have evolved to mitigate the impacts of spongy moth infestations. Spongy moth monitoring helps to control spongy moth outbreaks thereby improving the economic and environmental sustainability of spongy moth management and has some benefits for forest ecological niches [18]. In 2009, due to a severe outbreak of spongy moth larvae in the wooded regions on London, Ontario, Canada’s west side, forestry officials decided to implement a spray program for moth control [19]. The significance of assessing the ecological benefits and economic values of forests is particularly important [20], as forestry can have a significant impact on both the environment and human health [21].

The Whirlpool River Park is located in the southernmost deciduous forest region in Ontario, which comprises deciduous forest habitat at the southernmost point of the Niagara Escarpment along the Niagara River [22,23,24]. The selection of Whirlpool Forest for this study is of particular significance despite the vast number of forests across North America. Whirlpool Forest’s unique position at the southernmost point of the Niagara Escarpment renders it an exceptional ecological transition zone [25]. This geographical characteristic makes it an ideal sentinel site for monitoring the effects of climate change and invasive species on forest ecosystems. Furthermore, its proximity to Niagara Falls subjects it to distinct anthropogenic pressures, including high tourist traffic and urban encroachment [26]. This human–nature interface provides valuable insights into pest dynamics within human-impacted natural areas. The forest’s diverse tree species composition, especially its oak populations, offers a microcosm for studying spongy moth preferences and impacts across various host types. Additionally, the collection of long-term data from specific sites like Whirlpool Forest is crucial for understanding localized variations in pest behavior and for developing targeted, effective management strategies. While the findings from this study are specific to Whirlpool Forest, they contribute to a broader understanding of spongy moth ecology and can inform management practices in similar forest ecosystems across North America. This research thus bridges the gap between site-specific observations and broader ecological principles, offering valuable insights for forest management and conservation efforts. The study region has been widely used in ecology, geology, paleontology and other research [27,28]. However, previous research has primarily focused on the biological and ecological aspects of spongy moth invasions in North American forests [29,30]. There is a gap in the literature regarding the specific impacts on Whirlpool Forest and the local effectiveness of control strategies.

The objectives of this study are twofold, seeking to link localized ecological observations with broader projections of future spongy moth (*Lymantria dispar*) outbreaks in Ontario’s forests. At the local scale, the research examines infestation dynamics within the Whirlpool Forest by quantifying how tree species, size, and health influence the presence and abundance of egg masses, and by analyzing their vertical distribution to identify oviposition preferences and their implications for forest health and composition. At the broader scale, the study extends beyond the immediate study area by developing a climate-driven predictive model for Ontario, using Shared Socioeconomic Pathways (SSPs) to estimate potential outbreak area through 2100 and to evaluate the sensitivity of infestation patterns to changes in temperature and precipitation.

## 2. Materials and Methods

### 2.1. Study Area

**Figure 1:**
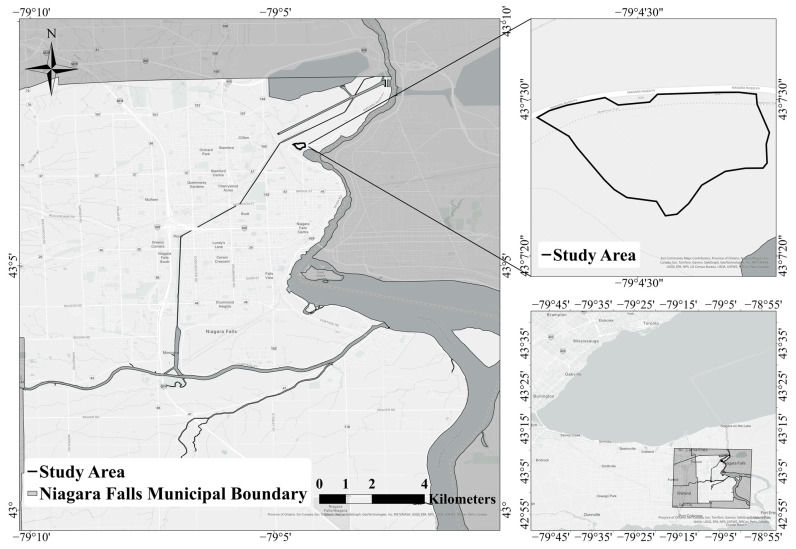
Map of the study area in the Whirlpool River Park, Niagara Falls, Ontario, Canada.

The study was conducted in the Niagara Gorge in eastern Canada. The study area (43°07′23″ N, 79°04′30″ W) was located within Whirlpool River Park in the province of Ontario (Figure 1). The Whirlpool River Park is situated at the southernmost point of the Niagara Escarpment, close to the Niagara River, and the predominant vegetation variety is deciduous. The region is regarded to have a moderate continental climate with consistent precipitation throughout the year [31]. The nearest climate station (Port Weller (AUT)) has an average March temperature of 2.3 °C and annual precipitation of 617.3 mm [32]. Sampling was conducted in two regions within Whirlpool Forest (near the intersection of Niagara Parkway and Whirlpool Road): (1) east, across from Legends Golf Course, one side near parking lot; (2) west, across from Legends Golf Course.

### 2.2. Field Data Collection

#### 2.2.1. Monitoring of Kaladar Plots (MKPs)

We drew on the methods described in “Gypsy (Spongy) Moth Egg Mass Surveys for the Woodlot Owner”, published by the Ontario Forest Health and Silviculture Section in 1999, as a guide to our methodology. We used Kaladar plots, a standardized 10 m × 10 m sampling method for forest pest surveys [33]. This technique is particularly effective for assessing spongy moth egg mass density. Eggs of the spongy moth (*Lymantria dispar*) were sampled during the daytime on March 25, 2022, using three distinct collection methods within each Kaladar plot: (1) sweep up fallen leaves to identify spongy moth eggs on the ground, (2) observation of spongy moth eggs in trees 0.1–1 m above ground level, and (3) using 10 × 42 binoculars (Nikon Inc., Melville, NY, USA) to observe spongy moth eggs on trees 1 m above the ground. All ground vegetation was sampled within the observable field of view of each Kaladar plot.

The study area was divided into two regions for multiple sampling according to standardized procedures: Multiple 10 m × 10 m Kaladar plots were set up at the study area and situated away from open spaces like roads or trails to prevent skewed counts. To establish the Kaladar plots within the woodlot, we advanced 20 m from the edge and used flagging tape to mark the initial corner. A diagonal line of 7.1 m was measured to find the plot’s center, which was then marked. Extending the line an additional 7.1 m from the center identified the opposite corner, ensuring the plot’s size at 10 m × 10 m. Lines drawn from the center to the two remaining corners, all marked with flagging tape, outlined each plot distinctly to facilitate accurate sampling and prevent overlap.

Sampling was conducted by sweeping inward along the edge of each Kaladar plot to record the number of all spongy moth eggs on the ground. Identification and counting of spongy moth eggs at heights of 0.1–1 m on the trees were initiated along the edges of Kaladar plots, and observations of spongy moth eggs above 1 m height on trees were started along the edge of Kaladar plots using binoculars. For each sample (defined as the number of spongy moth eggs collected at each 10 m × 10 m Kaladar plot), all spongy moth eggs were identified to distinguish between new and old eggs, with only new eggs counted. Distinguishing between new and old spongy moth egg masses is essential for accurate population surveys, with new egg masses being key for assessing current population levels [34]. The tree health analysis was based on a standardized dataset that pooled observations of spongy moth egg masses from 43 Kaladar plots across two regions of the study area. The 43 Kaladar plots were distributed across two regions of the Whirlpool Forest study area to ensure broad and representative coverage.

#### 2.2.2. Tree Identification and Calculation of Egg Masses

Tree identification involved analyzing morphological characteristics including leaf shape, size, and arrangement, bark texture and color, and the presence of flowers and buds. As the survey was conducted on 25 March 2022, most foliage observations came from fallen leaves and leaf buds. Priority was given to identifying tree species preferred by spongy moths, notably oaks (*Quercus* spp.) [16]. Field guides and digital databases were used for cross-referencing. Some oaks were classified as *Quercus* spp. due to the absence of diagnostic features during the winter survey period. In total, 157 trees representing 16 species were surveyed, including *Acer saccharum* (*n* = 39), *Quercus rubra* (*n* = 32), *Quercus macrocarpa* (*n* = 19), *Carya ovata* (*n* = 12), and others (see Table 1).

Spongy moth infestation was quantified by counting egg masses. We recorded 874 egg masses per 100 square meters on the forest floor, which was extrapolated to hectares using the formula $\frac{874\;\frac{egg\;masses}{100\;m^{2}}\times 100}{43}$. The results were compared with the Ministry of Natural Resources and Forestry Ontario’s pesticide spraying guidelines to assess outbreak severity. These guidelines indicate that 1250 egg masses per hectare (EM/ha) suggest light defoliation (up to 25%) in the following growing season. Moderate defoliation (26–65%) is expected at 1251–3750 EM/ha, heavy defoliation (66–90%) at 3750–5000 EM/ha, and severe defoliation (91–100%) at over 5000 EM/ha, assuming a healthy population and large egg masses. Defoliation levels above 65% can significantly stress trees, depleting starch reserves and hindering future growth. The raw data supporting these results are provided in Supplementary Material S1.

### 2.3. Data Analysis and Modeling

#### 2.3.1. Field Data Analysis

Data analysis was performed using Python 3.11.7. The study illustrated spongy moth distribution across tree species. A two-way ANOVA with Šídák’s multiple-comparisons test and paired *t*-tests compared egg mass densities above and below 1 m height. Correlation analysis used Spearman’s rank correlation to assess the relationship between tree diameter at breast height (DBH) and egg mass abundance at the two height levels. The Chi-square test of independence examined the association between tree health and egg mass presence (1 = presence, 0 = absence). Tree health was defined as unhealthy if two or more than two out of the following five parameters were abnormal: bark wounds, co-dominant crowns, unbalanced crowns, lack of annual growth, and dead branches.

#### 2.3.2. Historical Data Analysis and Regression Modeling

Historical climate data (2015–2023) for Ontario was obtained from ERA5-Land reanalysis, covering 41° N to 57° N and 265° E to 286° E. Area-weighted averaging was applied to account for grid cell size variations. Monthly precipitation data was converted to annual totals (mm), and temperature data was converted from Kelvin to Celsius and averaged annually. Annual spongy moth outbreak area data (hectares) was collected from annual Ontario government forest health conditions reports [11,35,36,37,38,39,40,41].

Using this data, we developed an Ordinary Least Squares (OLS) regression model using Python 3.11.7 to examine the relationship between outbreak area and environmental factors:

$$\begin{array}{ll} Outbreak\;Area & =\beta_{0}+\beta_{1}\left(Total\;Annual\;Precipitation\right)\\ & +\;\beta_{2}\left(Average\;annual\;temperature\right)\\ & +\;\varepsilon \end{array}$$

where *β*_0_ is the intercept, *β*_1_ and *β*_2_ are coefficients for total precipitation and average temperature, respectively, and ε is the error term. The regression analysis was based on 9 annual observations from 2015 to 2023. Although the sample size is relatively limited, it is consistent with the temporal resolution of the outbreak records and comparable to other ecological time-series studies [42,43,44,45].

#### 2.3.3. Model Validation and Selection

To validate our model and ensure its robustness, we employed several statistical techniques. We examined the model’s overall fit using the coefficient of determination (*R^2^*) and adjusted *R^2^*, which indicated the proportion of variance in the outbreak area (dependent variable) predictable from temperature and precipitation (independent variables). T-statistics and *p*-values were analyzed for individual variable significance. Residual analysis checked linear regression assumptions through visual inspection of residual and normal probability plots. Multicollinearity was assessed using the Variance Inflation Factor (VIF) for each predictor, with values substantially greater than 5 indicating potential multicollinearity issues.

#### 2.3.4. Future Projections and Sensitivity Analysis

Using climate projections for different Shared Socioeconomic Pathways (SSPs) scenarios in 2100, we applied our selected model to predict potential outbreak area. We considered SSP1-2.6 (sustainability), SSP2-4.5 (middle of the road), SSP3-7.0 (regional rivalry) and SSP5-8.5 (fossil-fueled development) scenarios.

Our sensitivity analysis focused on understanding how variations in temperature and precipitation influence outbreak predictions under each SSP scenario. We varied these parameters within projected ranges to assess their impact on predicted outbreak area.

## 3. Results

### 3.1. Impact of Spongy Moths on Different Tree Species

The distribution of spongy moth egg masses at different heights on various tree species was quantified at 43 sampling plots in the Whirlpool Forest, Ontario, Canada. A notable concentration of egg masses was observed above 1 m in several species, indicating a potential preference or accessibility factor for egg-laying moths at this height. The result showed that oaks (*Quercus* spp.) at a height of more than 1 m were the species most infested with spongy moth eggs (Table 2). The most heavily infested species, in terms of total egg masses recorded across all surveyed individuals, were red oak (*Quercus rubra*) with 381 egg masses, bur oak (*Quercus macrocarpa*) with 138 egg masses, and an unidentified oak species with 78 egg masses. This third oak species, while clearly belonging to the Quercus genus, could not be definitively identified to the species level during our survey. Similar results can also be seen in spongy moth egg infestations below 1 m. The most heavily infested tree species continue to be oaks. They were red oak, bur oak, sugar maple (*Acer saccharum*), white oak (*Quercus alba*), and oak, with spongy moth egg counts of 64, 21, 19, 16, and 12, respectively. Tree infestation status is summarized in Table 1, which shows that *Quercus* spp. also had the highest proportion of infested individuals among surveyed species, further confirming that oaks are the most at-risk species in the study region.

### 3.2. Spongy Moth Egg Masses on Trees Above and Below 1 m

A comparison of egg mass abundance across tree species and oviposition heights (>1 m vs. <1 m) revealed strong species-level differences. The two-way ANOVA showed that both the main effect of tree species (*p* < 0.001) and the interaction between species and height (*p* = 0.018) were significant, together explaining 21.03% of the total variation. In particular, the interaction term (7.76%) indicated that certain tree species were more prone to height-dependent infestation. By contrast, the main effect of height alone was not significant (*p* = 0.066), suggesting that spongy moths do not show a uniform preference for laying eggs above or below 1 m when considering all species together (Table 3).

Post hoc Šídák’s multiple comparisons clarified which species contributed to these effects (Table 4). Only red oak (*Quercus rubra*) showed a significant difference between heights, with egg masses above 1 m being markedly higher than below (*p* < 0.001). Other species, including bur oak (*Q. macrocarpa*), white oak (*Q. alba*), sugar maple (*Acer saccharum*), and white ash (*Fraxinus americana*), exhibited no significant differences, although several displayed a trend toward higher values above 1 m. These results indicate that the general absence of a main height effect masks important species-specific patterns, with red oak being uniquely susceptible to elevated oviposition.

At the whole-forest level, a paired *t*-test comparing mean egg mass counts above and below 1 m across the 16 surveyed species yielded no significant overall difference (*t*(15) = 1.703, *p* = 0.109). The mean difference was –34.88 egg masses, with a wide 95% CI (–78.53 to 8.78), reflecting considerable variability in infestation among species. Despite this variability, the strong correlation coefficient between paired values (*r* = 0.946, *p* < 0.001) confirmed that the dataset was internally consistent and that the observed variation is more likely biological than methodological.

### 3.3. Relationship Between DBH and Spongy Moth Egg Masses

**Figure 2:**
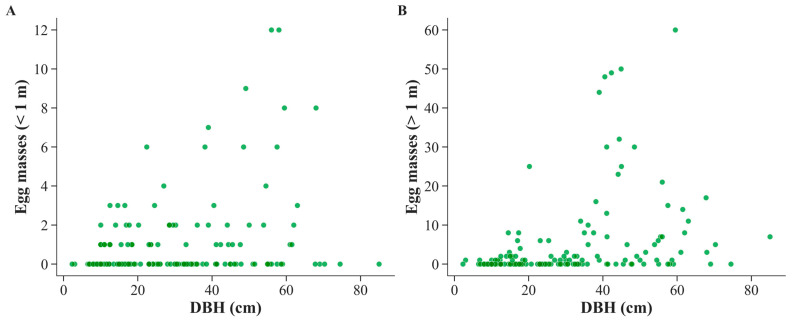
Relationship between tree diameter at breast height (DBH) and spongy moth (*Lymantria dispar*) egg masses across all surveyed tree species. (**A**) Egg masses deposited below 1 m. (**B**) Egg masses deposited above 1 m.

The relationship between tree diameter at breast height (DBH) and spongy moth oviposition was analyzed using Spearman’s rank correlation to account for non-linear variation in the data (Figure 2). Across all species (*n* = 157, including non-infested trees), egg abundance showed a significant positive correlation with DBH both below 1 m (*ρ* = 0.218, *p* = 0.006) and above 1 m (*ρ* = 0.458, *p* < 0.001). These results indicate that larger trees generally host more eggs, with the relationship particularly strong above 1 m.

**Figure 3:**
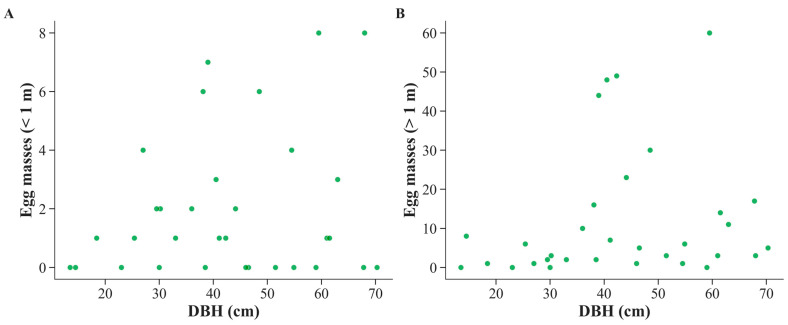
Relationship between tree diameter at breast height (DBH) and spongy moth (*Lymantria dispar*) egg masses in red oak (*Quercus rubra*) at two height classes. (**A**) Egg masses deposited below 1 m. (**B**) Egg masses deposited above 1 m.

When focusing on the dominant host, red oak (*Quercus rubra*; *n* = 32), no significant relationship was detected below 1 m (*ρ* = 0.096, *p* = 0.600), but a positive trend was observed above 1 m (*ρ* = 0.339, *p* = 0.058), suggesting a tendency for greater oviposition on taller portions of larger individuals (Figure 3). The lack of significance below 1 m may reflect greater stochasticity in oviposition near the ground, where litter cover, microclimate, and predation pressure can obscure the influence of DBH. Although not statistically significant at the 0.05 level, the above-1 m trend is consistent with the whole-forest pattern and supports the interpretation that red oak is particularly vulnerable to oviposition higher on the trunk.

### 3.4. Number of Healthy Trees in Relation to Egg Masses

The results of the chi-square test of independence are summarized in Table 5, which cross-classifies trees by egg mass presence (1 = presence, 0 = absence) and health status (1 = healthy, 0 = unhealthy). The dataset included 157 trees, with expected frequencies calculated for each category. Trees were categorized based on egg mass presence and health parameters, including bark wounds, canopy structure, growth, and dead branches. The analysis yielded a chi-square statistic of 6.08 (*df* = 1, *p* = 0.014), indicating a statistically significant association between the presence of spongy moth egg masses and tree health.

### 3.5. Historical Climate Analysis and Regression Modeling

The OLS regression model explained 71.4% of the variance in spongy moth outbreak area (*R^2^* = 0.714, Adjusted *R^2^* = 0.600, F(2,5) = 6.247, *p* < 0.05). Total annual precipitation was a marginally significant predictor (*β* = 2590.7661, *t* = 2.490, *p* = 0.055), while average annual temperature showed a significant relationship (*β* = 814,100, *t* = 3.522, *p* < 0.05). The model can be expressed as:

Outbreak Area = −5,140,000 + 2590.7661 (Total Annual Precipitation) + 814,100 (Average Annual Temperature)

The variance inflation factor (VIF) values for both total annual precipitation and mean annual temperature were 1.67. These low VIF values are well below the common thresholds of 5 or 10, suggesting that multicollinearity is not a significant issue in the model. The Durbin–Watson statistic of 1.942 suggests no significant autocorrelation in the residuals. The Jarque–Bera test (JB = 0.469, *p* = 0.791) indicates that the residuals are normally distributed.

### 3.6. Future Projections and Sensitivity Analysis

**Figure 4:**
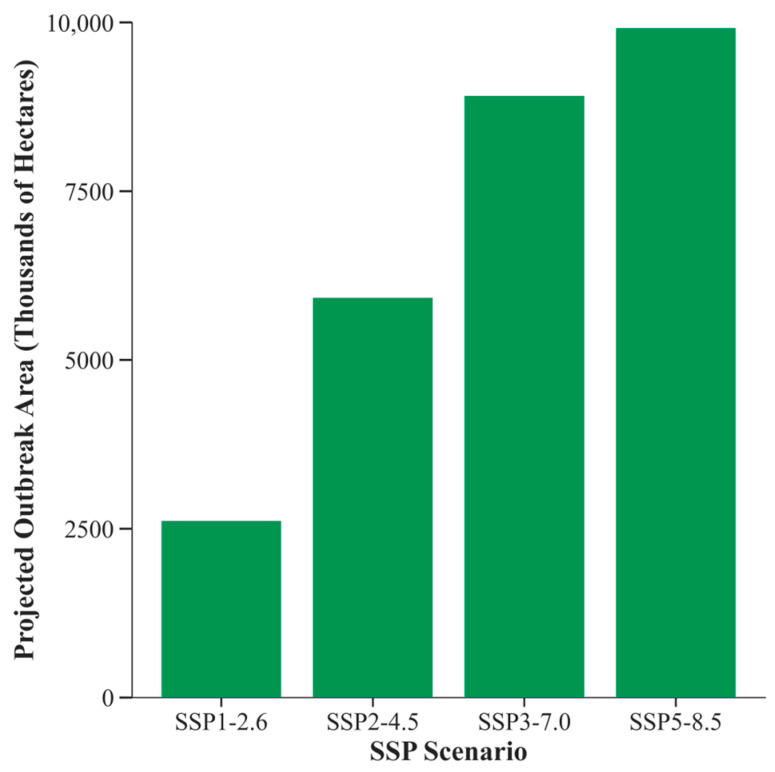
Projected outbreak area of spongy moth (*Lymantria dispar*) in Ontario under four Shared Socioeconomic Pathways (SSP1-2.6, SSP2-4.5, SSP3-7.0, SSP5-8.5) by 2100. Outbreak area is expressed in hectares.

We applied our model to climate projections for different SSP scenarios for 2100 (Figure 4). The projected outbreak area varied significantly: 2,625,819.89 hectares under SSP1-2.6, 5,931,532.84 hectares under SSP2-4.5, 8,921,692.37 hectares under SSP3-7.0, and 9,927,378.49 hectares under SSP5-8.5. These results demonstrate a nearly four-fold increase from the best- to worst-case scenarios.

Sensitivity analyses revealed that small climatic changes could significantly expand the outbreak area. A 1 °C increase in mean annual temperature could expand the outbreak area by 814,100 hectares, while a 100 mm increase in annual precipitation may add 25.907661 hectares.

## 4. Discussion

### 4.1. Impacts of Spongy Moth on Forest Species and Health

Our findings on spongy moth preferences and behavior in Whirlpool Forest, Ontario, largely align with existing knowledge. The observed preference for oak species, particularly red oaks, corroborates previous studies [46,47]. Species composition significantly affects defoliation outcomes, with oak-dominated stands experiencing higher mortality rates [46,47]. Our research supports Gansner et al.’s [16] observations on species-specific preferences, likely due to variations in foliage quality and habitat conditions. Inoue et al. [48] emphasize the importance of understanding pest-host dynamics for effective management, suggesting a need for targeted, species-centric strategies [49].

The distribution of egg masses reveals a nuanced pattern related to tree height. Thorpe and Ridgway [50] found that egg masses favor certain tree species and heights, possibly due to reduced predation risk or favorable microclimatic conditions [51,52]. Our findings of higher egg mass counts above 1 m might indicate lower population densities [53], though further research is needed. This observation underscores the complexity of spongy moth behavior and the importance of considering multiple factors in management strategies.

Our analysis revealed a statistically significant but weak positive correlation between tree diameter and spongy moth egg mass density. Cook, Hain, and Smith [54] reported a similar preference, noting that larger trees may provide more resources or suitable microhabitats for spongy moth survival. This suggests that tree diameter, as a proxy for size and age, plays a crucial role in forest pest dynamics, potentially influencing outbreak susceptibility [55,56]. However, this relationship warrants cautious interpretation, as higher egg mass counts on larger trees may primarily result from increased surface area rather than higher susceptibility per unit area. The apparent preference of spongy moths for larger trees indicates an opportunistic resource exploitation approach, highlighting the importance of tree age and growth stages in forest management. This finding suggests potential strategies to manage younger forests distinctively and introduces the prospect of strategic forest thinning and diversification as preemptive measures against infestations.

The weak correlation between egg mass presence and tree health aligns with Davidson et al.’s [46] findings on defoliation stress. Scriber [57] and Wagner and Van Driesche [58] emphasize the complex relationship between pest presence and ecological health, suggesting the need for a multifaceted approach to pest management [59]. This complexity underscores the necessity of moving beyond simple pest eradication strategies towards holistic forest management that prioritizes both ecosystem health and pest control.

### 4.2. Egg Mass Density and Defoliation Risk Assessment

The calculated 2033 egg masses per hectare in our study area falls within the “Moderate” defoliation forecast category according to the Ministry of Natural Resources and Forestry Ontario’s guidelines. This indicates an expected defoliation range of 26% to 65% in the upcoming growing season, suggesting a significant but not critical threat to tree health and canopy integrity. While this level of egg mass density signals a considerable risk of noticeable defoliation, it does not warrant the most severe management responses.

Given this “Moderate” classification, immediate implementation of pesticide spraying may be premature. Alternative, non-chemical management strategies should be considered first, such as encouraging natural predators, using burlap bands to trap caterpillars, and promoting community-based egg mass removal efforts. These approaches align with the guidelines provided by the Ministry and offer potentially effective ways to manage the infestation without resorting to chemical interventions.

### 4.3. Climate Change and Spongy Moth Outbreaks

Our model explains 71.4% of the variance in spongy moth outbreak area, highlighting the significant impact of climate variables on forest pest dynamics. The marginally significant positive correlation between total annual precipitation and outbreak area suggests that increased moisture enhances egg survival and larval development, possibly due to improved food quality and reduced desiccation risk. The significant positive effect of average annual temperature on outbreak area corroborates findings from other studies indicating that warmer temperatures can accelerate spongy moth development and potentially increase the number of generations per year [60,61,62]. Higher temperatures may also expand the geographical range suitable for spongy moth establishment, potentially leading to larger outbreak area.

### 4.4. Future Projections and Management Implications

The projected spongy moth outbreak area under different SSP scenarios for 2100 presents a concerning outlook for Ontario’s forest management. Even the most optimistic scenario (SSP1-2.6) predicts a substantial increase to 2,625,819.89 hectares, while the worst-case scenario (SSP5-8.5) projects an alarming 9,927,378.49 hectares potentially affected. These projections emphasize the urgent need for adaptive management strategies that account for changing climate conditions.

Current spongy moth management employs an integrated approach, combining biological insecticides for high population densities with mating disruption using synthetic female pheromones for low densities along the range front [63]. Bacillus thuringiensis (Btk) is the primary agent for suppressing high-density populations [64], while Gypchek is used in sensitive areas [65,66]. Chemical insecticides have been largely phased out for broad forest use, especially on public lands [67]. In light of these projections, forest managers should consider enhancing monitoring systems for more accurate outbreak predictions, continuing research into biological control methods, implementing landscape-level planning those accounts for forest composition and climate change projections, and developing adaptive management approaches that can swiftly respond to changing infestation patterns. These strategies should be integrated with existing successful practices to create comprehensive, sustainable forest management plans. The sensitivity analysis indicates that relatively small shifts in climate variables can lead to significant changes in outbreak area. This underscores the importance of continuous monitoring and adaptive management approaches that can respond to ongoing climate changes. Forest managers must remain vigilant and ready to adjust their strategies based on new climate data and predictive models. This flexible, forward-thinking approach will be crucial in addressing future challenges posed by spongy moth outbreaks in a changing climate.

### 4.5. Integration of Ecological and Climate Modeling Insights

Integrating species-specific vulnerabilities, egg mass distribution patterns, and climate modeling results provides a comprehensive view of future spongy moth dynamics in Ontario forests. The preference for oak species, coupled with projected climate changes, suggests oak-dominated forests may face increased risk, necessitating prioritized management strategies. The relationship between tree diameter and egg mass density, considered alongside climate projections, indicates mature oak stands may become more susceptible to outbreaks, emphasizing the need for age-diverse forest management and potentially more frequent thinning. Additionally, the weak correlation between egg mass presence and tree health, in the context of changing climate, suggests a complex interaction between pest pressure and tree resilience, underscoring the need for proactive management strategies that consider both current tree health and future climate scenarios.

### 4.6. Research Limitations and Prospections

Our study on spongy moth infestations in Whirlpool Forest, Ontario, while providing important insights, has several limitations that inform future research directions. The confined geographical scope may restrict the generalizability of our findings to other regions. Our predictive model faced challenges in capturing long-term cyclical patterns of spongy moth populations. Attempts to extend the temporal range of data led to a significant decrease in model fit, indicating the complexity of factors influencing outbreaks. While we considered additional climate variables in the model, their impact was minimal, and some exhibited counterintuitive correlations. The absence of data on management interventions, such as aerial spraying, introduces potential variations unaccounted for in the model. Furthermore, the use of area-weighted average climate data may obscure local climate variations that could affect spongy moth dynamics.

Future research should expand to include a broader range of forest types and ecosystem interactions. We recommend developing more detailed, quantitative measures of tree health to better understand how tree vitality influences susceptibility to infestations. To improve predictive modeling, future studies should explore non-linear techniques and machine learning algorithms to reveal possible indirect or complex effects of climate variables. Incorporating management intervention data and utilizing higher-resolution climate data could enhance model precision. A multidisciplinary approach integrating entomology, forest ecology, climatology, and data science would yield deeper insights into spongy moth dynamics and inform adaptive management strategies.

## 5. Conclusions

This study provides a comprehensive analysis of spongy moth (*Lymantria dispar*) infestations in Whirlpool Forest, Ontario, integrating local ecological observations with future climate projections. Key findings reveal that spongy moths show a strong preference for oak species, particularly red oaks, with egg masses concentrated above one meter in height. Predictive modeling indicates a substantial increase in outbreak-prone area by 2100, even under optimistic climate scenarios. Notably, small variations in temperature and precipitation significantly influence outbreak area, highlighting forest ecosystem vulnerability to climate change.

These findings offer valuable guidance for forest managers and policymakers. Management strategies should focus on species-specific vulnerabilities and incorporate future climate projections into planning. There is a clear need to develop adaptive approaches that respond to evolving environmental conditions, potentially combining traditional management techniques with innovative strategies derived from predictive modeling.

This research contributes to the broader field of forest pest management by demonstrating the importance of integrating localized ecological studies with climate modeling. It provides a robust framework for understanding the complex interactions between forest ecosystems, invasive species, and climate change, informing future research and management efforts. By bridging local empirical data with large-scale climate projections, the study offers critical insights for both immediate forest management practices and long-term strategic planning in the face of changing environmental conditions.

## Figures and Tables

**Table 1:** Tree infestation status by spongy moth (*Lymantria dispar*) in Whirlpool River Park, Niagara Falls, Ontario, 2022. Values represent the number of trees observed per species classified as infected (egg masses present) or not infected.

Tree Species (Scientific Name)	Not Infected	Infected	Total
*Quercus rubra* (Red oak)	1	31	32
*Acer saccharum* (Sugar maple)	12	27	39
*Quercus macrocarpa* (Bur oak)	2	17	19
*Quercus* spp. (Oak)	1	7	8
*Tilia americana* (Basswood)	2	5	7
*Acer rubrum* (Red maple)	2	4	6
*Fraxinus americana* (White ash)	3	4	7
*Quercus alba* (White oak)	2	4	6
Unknown	0	3	3
*Carya cordiformis* (Bitternut hickory)	0	2	2
*Ostrya virginiana* (Ironwood)	1	2	3
*Acer* spp. (Maple)	3	2	5
*Carya ovata* (Shagbark hickory)	10	2	12
*Fraxinus* spp. (Ash)	0	1	1
*Fraxinus pennsylvanica* (Green ash)	6	0	6
*Acer saccharinum* (Silver maple)	1	0	1

**Table 2:** Spongy moth (*Lymantria dispar*) egg mass distribution above and below 1 m on different tree species in Whirlpool River Park, Niagara Falls, Ontario, 2022. Values represent the number of egg masses recorded for each species.

Tree Species (Scientific Name)	Above 1 m	Below 1 m	Total
*Quercus rubra* (Red oak)	381	64	445
*Quercus macrocarpa* (Bur oak)	138	21	159
*Quercus* spp. (Oak)	78	12	90
Unknown	37	3	40
*Acer saccharum* (Sugar maple)	27	19	46
*Fraxinus americana* (White ash)	25	9	34
*Ostrya virginiana* (Ironwood)	11	1	12
*Acer rubrum* (Red maple)	8	3	11
*Quercus alba* (White oak)	6	3	9
*Carya ovata* (Shagbark hickory)	16	3	19
*Carya cordiformis* (Bitternut hickory)	1	2	3
*Acer* spp. (Maple)	4	1	5
*Tilia americana* (Basswood)	1	1	2
*Fraxinus* spp. (Ash)	2	0	2
*Fraxinus pennsylvanica* (Green ash)	0	0	0
*Acer saccharinum* (Silver maple)	0	0	0

**Table 3:** Two-way ANOVA results on the effects of tree species (row factor) and oviposition height (column factor: above vs. below 1 m) on the number of spongy moth egg masses.

Source of Variation	% of Total Variation	DF	MS	F(DFn, DFd)	*p* Value	Significance
Interaction	7.76	15	96.06	F(15, 282) = 1.96	0.018	*
Tree species (Row)	12.37	15	153.10	F(15, 282) = 3.12	<0.001	***
Height (Column)	0.90	1	167.60	F(1, 282) = 3.42	0.066	ns
Residual	79.00	282	49.08	—	—	—

Notes: “—” = not applicable; “ns” = not significant; * *p* < 0.05; *** *p* < 0.001.

**Table 4:** Šídák’s multiple comparisons of spongy moth egg masses between oviposition heights (Above vs. Below 1 m) within each tree species.

Tree Species	Mean Diff. (Below − Above)	95% CI of Diff.	Adjusted *p* Value	Significance
*Quercus rubra* (Red oak)	–9.91	–15.11 to –4.70	<0.001	***
*Quercus macrocarpa* (Bur oak)	–6.16	–12.92 to 0.60	0.109	ns
*Quercus* spp. (Unidentified)	–11.33	–28.34 to 5.68	0.549	ns
*Quercus alba* (White oak)	+1.67	–10.36 to 13.69	>0.999	ns
*Acer saccharum* (Sugar maple)	–0.21	–4.92 to 4.51	>0.999	ns
*Fraxinus americana* (White ash)	–2.00	–13.13 to 9.14	>0.999	ns
*Carya ovata* (Shagbark hickory)	–0.17	–8.67 to 8.34	>0.999	ns
All other species	ns	—	—	ns

Notes: “—” = not applicable; “ns” = not significant; *** *p* < 0.001.

**Table 5:** Contingency table of spongy moth egg mass presence and tree health status in Whirlpool Forest. Tree health was classified as unhealthy (1) if two or more than two of the following parameters were abnormal: bark wounds, co-dominant crowns, unbalanced crowns, lack of annual growth, and dead branches.

Egg Masses (Presence/Absence)	Unhealthy (0)	Healthy (1)	Total
Absence (0)	23	23	46
Presence (1)	80	31	111
Total	103	54	157

Notes: Chi-square test of independence: *χ^2^* = 6.08, *df* = 1, *p* = 0.014.

## Data Availability

All the data analyzed or generated during this study are included in this published article and Supplementary information files.

## Supplementary Materials

The following supporting information can be downloaded at: https://www.mdpi.com/article/10.3390/biology14111506/s1. Table S1: Forest Health data NPCWhirlpool Forest.csv/s1.
